# Validation of intensive care unit predictive scoring systems in West Africans

**DOI:** 10.1016/j.mex.2026.103817

**Published:** 2026-02-09

**Authors:** Charles Frederick Hayfron-Benjamin, Theresa Ruby Quartey-Papafio, Akua Kissi-Prah, Delphine Delali Grant, Tracy Amo-Nyarko, Anastasia Naa Koshie Bruce, Divine Agbenyegah Kwami, Andrew Kwabena-Adade

**Affiliations:** a Department of Physiology, University of Ghana Medical School, P O Box GP 4236, Accra, Ghana; b Department of Anesthesia and Critical Care, Korle Bu Teaching Hospital, P. O. Box 77, Korle Bu, Accra, Ghana; c Department of Anesthesia and Critical Care, University of Ghana Medical Centre, Ghana; d Department of Anesthesia and Critical Care, Greater Accra Regional Hospital, Ghana; e Departments of Vascular Medicine, Respiratory Medicine, and Public Health, Amsterdam UMC, University of Amsterdam, the Netherlands

**Keywords:** Predictive scoring systems, Intensive care unit, Critical care, Sub-Saharan Africans

## Abstract

Intensive care unit(ICU) predictive scoring systems (PSS) are valuable in predicting outcomes in the ICU. However, they have not been validated in many populations, including West Africans, limiting their utility in these populations. The study population comprises Ghanaians managed in the ICUs of two major tertiary/quaternary hospitals in Ghana from 2017 to 2026. The Acute Physiologic and Chronic Health Evaluation-IV(APACHE-IV), Simplified Acute Physiologic Score-III (SAPS-III), Mortality Prediction Model-III(MPM0-III), Sequential (Sepsis-related) Organ Failure Assessment(SOFA)/quick SOFA(qSOFA), and National Early Warning Score-2(NEWS-2) scores will be calculated. For APACHE-IV, SAPS-III, and MPM0-III, the primary outcome measures are mortality and/or length of stay(LOS). The APACHE IV, SAPS-III, and MPM0-III scores and their corresponding predicted mortality ratios will be calculated. The overall observed and predicted ICU mortality rates will be assessed. Validation will be tested by assessing calibration and discrimination. For SOFA/qSOFA and NEWS-2, the associations of the mean score, highest score, and change in score with mortality will be assessed. Conventional statistical tools and supervised machine learning algorithms will be used to identify predictors of ICU mortality.

• This study aims to validate the performance of existing ICU PSS in predicting ICU mortality and LOS among West Africans, and to identify their predictors.

## Graphical abstract

**Figure:**
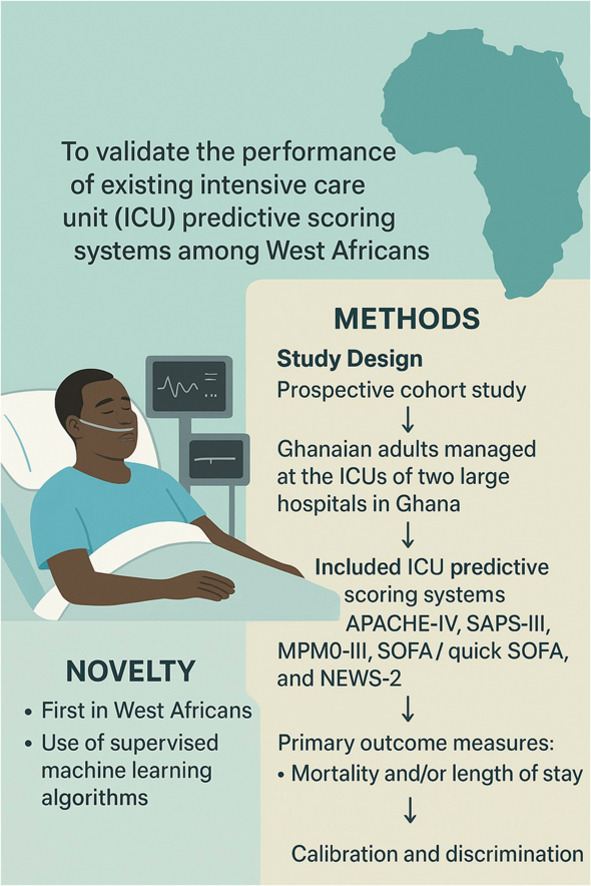

**Specifications table**

**Table:** 

**Subject area**	Medicine and Dentistry
**More specific subject area**	Intensive care medicine and emergency medicine
**Name of your protocol**	Validation of intensive care unit predictive scoring systems in West Africans
**Reagents/tools**	N/A
**Experimental design**	N/A
**Trial registration**	N/A
**Ethics**	The study is approved by the Scientific and Technical Committee and Institutional Review Board of the KBTH (Number: KBTH-IRB 000135/2022) and the University of Ghana College of Health Sciences Ethical and Protocol Review Committee (CHS-Et.M.1-P4.13/2023-2024).
**Value of the Protocol**	• To our knowledge, this proposed study will be the first to validate all major intensive care unit predictive scoring systems in a sub-Saharan African population. • An additional value of this study is the use of machine learning algorithms in identifying predictors of ICU outcomes, including mortality and length of stay.

## Background

Intensive care unit(ICU) Predictive scoring systems(PSSs) are important adjuncts for improving clinical decision-making and standardizing ICU research [[1], [2], [3]]. Commonly used ICU PSSs in the assessment of severity of disease at ICU admission and prediction of in-hospital mortality include versions of the Acute Physiologic and Chronic Health Evaluation (APACHE) scoring system [4], Simplified Acute Physiologic Score(SAPS) [5], and the Mortality Prediction Model (MPM0) [6]. ICU PSSs for severity assessment over ICU stay include the Sequential (Sepsis-related) Organ Failure Assessment [SOFA] [7], quick SOFA (qSOFA) [8], and National Early Warning Score (NEWS) [9]. SOFA/qSOFA [10,11] and NEWS [9,12] scores and changes in their scores are known to correlate with in-hospital mortality.

The existing ICU PSSs are based on data from patients managed in the ICUs of high-income countries [4]. Existing data show important ethnic differences in ICU outcomes, including mortality [13]. In explaining such differences, structural inequalities in care have been cited as the likely underlying reason [13]. However, disparities in the performance of predictive equations assessing disease severity, which in turn influences treatment, cannot be ruled out [14]. A study by Sarkar et al. comparing the performance of ICU PSSs in different ethnicities shows important ethnic differences. For example, the APACHE IV and SOFA scores over-predicted mortality in African American and Hispanic groups compared with non-Hispanic whites [15]. Apart from ethnicity, environmental factors influence several physiological variables, including disease susceptibility [[16], [17], [18], [19]].

To date, ICUs in many parts of the world, including West Africa, do not utilize PSSs, as they have not been validated in these populations. ICU PSSs perform best in the population they were derived from and using them in a population in which it has not been validated may result in poor performance [14]. To apply the existing ICU PSS in a West African population, they must first be validated in that population, and a customized equation that works best for the population be developed to improve performance. There is, therefore, an urgent need to validate the already existing ICU PSSs in West Africans and to develop a customized equation that works best for the population. Another key limitation of the existing ICU PSS is that they were developed in a period that lacked computation methods like machine learning (ML) capable of analyzing large datasets to identify patterns and complex relationships, where the traditional methods are very limited [20]. This can result in a more efficient prediction of outcomes, including mortality.

To address the above gaps, the proposed study has two objectives. First, it seeks to validate the performance of the existing ICU PSS in a West African population. To achieve this, the major PSS used in predicting mortality in general ICU patients will be assessed in patients managed in the ICUs of two large referral hospitals in Ghana. In the primary analysis, the current versions of these scores (i.e, APACHE-IV, SAPS-III, and MPM0-III) will be evaluated. For SOFA/qSOFA and NEWS-2, the associations of the mean score, the highest score, and the change in score with mortality will be assessed. The second objective is to employ ML algorithms to identify predictors of these outcomes in Ghanaian adults.

## Description of protocol

### Study design and setting

This is an investigator-initiated, longitudinal observational cohort study involving the surgical (general surgical, obstetric, burns, and trauma), medical, and cardiothoracic ICUs at two major tertiary/quaternary hospitals in Ghana, namely the Korle Bu Teaching Hospital (KBTH) and the University of Ghana Medical Centre (UGMC). On average, the included ICUs care for over 400 patients each year. KBTH is the largest and leading tertiary referral center in Ghana and the third largest in Africa. KBTH attracts referral cases from all over Ghana. The UGMC is the first and currently the only quaternary-level healthcare provider in Ghana. The KBTH and UGMC ICUs provide critical care for patients referred from departments within the hospital as well as many hospitals in the country. The ICUs have equipment for invasive and non-invasive mechanical ventilation and physiological monitoring, and for the measurement of arterial blood gases. The ICUs are run by multidisciplinary care teams including anesthesiologists/intensivists, physicians/surgeons, and critical care nurses. The ICUs also receive support from relevant specialties such as physiotherapists and nutritionists/dieticians in the hospital. These study findings will be reported according to the STrengthening the Reporting of OBservational studies in Epidemiology (STROBE) statement.

### Eligibility criteria and recruitment process

Data to be included are from patients managed in the included ICUs from January 2017 to March 2026. Eligible participants are Ghanaians aged 18 years and above without a recent ICU admission and requiring ICU care. Ghanaian ethnic background will be determined by verifying the genealogy of the participant up to the second generation (grandparents) using a nativity questionnaire. A clinical abstraction form will be used to capture relevant data (needed to calculate the included scores and outcome measures) from the medical records. For patients with two or more admissions to the ICU during the same hospital stay, only the data from the first admission will be included in the analyses.

The ethics boards approved the inclusion of data from participants managed in the ICUs before the study was approved by the ethics board. For participants included after the study was approved by the ethics boards, informed consent was required before abstraction of their data from the medical records. After explaining the study purpose and procedure to each participant/surrogate decision-maker, informed consent will be sought. Patients will only be recruited into the study when they (if adequately capacitated) or their surrogate decision-makers give voluntary, competent, and appropriate informed consent. In addition to retrieving relevant data from the medical records, biological samples will be taken for storage and future analyses of biomarkers.

A screening log will be completed by the principal investigator to provide documentation of all records retrieved. The screening log will include the following information: 1) study identification number, 2) participant’s name, age, sex, and contact details (and the name and contact details of the surrogate decision-maker, where applicable), 3) date informed consent was obtained (where applicable) and 4) participant’s eligibility status for the study. The screening log would be updated every week by the principal investigator until the recruitment is completed. The screening log will be kept at the study site only to act as the master list for linking participants to protected health information.

### Measurements

A clinical abstraction form will be used to extract relevant data required to calculate the various ICU PSS scores. The data to be collected are summarized in Table 1. They include sociodemographic, previous/current clinical health information, physiological variables, and laboratory measurements of study participants will be used to calculate numerical severity of illness scores. Baseline measurements within one hour of admission and within 24 hours of admission will be collected. The frequency of repeat measurements to be collected will be guided by the specific ICU PSS. For APACHE-IV and SAPS-III, the worst physiologic values measured within 24 hours of admission will be used. For MPM0-III, data within one hour of ICU admission will be used. For SOFA/qSOFA, data collected 24 hours after admission and every 48 hours thereafter will be used. For the NEWS-2 score, data collected will be at admission, and every 12 hours (for NEWS-2 score 0) or 4–6 hours (for NEWS-2 scores of 1–4). The APACHE IV score, APS (Acute Physiology Score), estimated mortality rate, and estimated LOS will be calculated using a calculator available at https://intensivecarenetwork.com/Calculators/Files/Apache4.html. Ts SAPS-III score will be calculated using a calculator available at https://www.mdcalc.com/calc/10403/simplified-acute-physiology-score-saps-3. The MPM00-III score will be calculated using a calculator available at https://intensivecarenetwork.com/Calculators/Files/Mpm2.html.The SOFA score will be calculated using a calculator available at https://www.mdcalc.com/calc/691/sequential-organ-failure-assessment-sofa-score. The qSOFA score will be calculated using a calculator available at https://www.mdcalc.com/calc/2654/qsofa-quick-sofa-score-sepsis. The NEWS score will be calculated using a calculator available at https://www.mdcalc.com/calc/1873/national-early-warning-score-news. ICU length of stay (LOS) in days will be defined as the time from admission to discharge from the ICU. At the KBTH ICUs, discharge from the ICU is generally indicated if patients attain physiological stabilization (including stable hemodynamic measures and other vital signs and not requiring invasive hemodynamic monitoring or life organ supports available at the ICUs), reasonable resolution of the acute illness or reason for admission to ICU, and the ability to be managed at a lower level.

**Table 1:** Variables to be extracted from medical records.

Item	Data to be retrieved
Medical Record Number	
Date of Data Abstraction	____ / ____ / _______ (MM/DD/YYYY)
1. Type and location of ICU	____ / ____ / _______ (MM/DD/YYYY)
2. Date and time of ICU admission	____ / ____ / ________ ________ (MM/DD/YYYY) (HH/MM)
3. Date and time of ICU discharge or death	____ / ____ / ________ ________ (MM/DD/YYYY) (HH/MM)
4. Location before ICU admission	Other ICUs, emergency room, other acute care facility, operating theatre, recovery ward, general ward, chronic care facilities, etc.
5. Length of stay in the care location before ICU admission	Dates of admission and transfer to the ICU.
6. Admission scheduling status	Whether ICU admission was planned/scheduled or not.
7. Previous ICU admissions	Previous ICU admissions or not. If yes, specify the indication for ICU admission, dates of admission and discharge.
8. Sociodemographic characteristics	Age, sex, marital status, and socioeconomic status (highest level of education and income).
9. Reason for referral to the ICU	Reason for referral as stated by the referring physician.
10. Medical history	Presenting complaints; history of presenting complaints; past medical history; medication history; known allergies; and family history.
11. Lifestyle	Smoking and alcohol consumption.
12. Chronic Health Conditions	Comorbid conditions, including chronic kidney disease, chronic heart failure, cirrhosis, hepatic failure, cancer (type to be specified), metastatic carcinoma, cancer therapy, lymphoma, leukemia or myeloma, other hematologic malignancies, immunosuppression, AIDS, and other conditions.
13. Other comorbidities	Sickle cell disease, asthma, diabetes mellitus, hypertension, and cardiovascular disease.
14. Acute conditions at presentation	Acute kidney injury, acute heart failure, cerebrovascular accidents/transient ischemic attack, acute coronary syndrome, cardiac dysrhythmias, pneumonia, gastrointestinal bleeding, and intracranial mass effect.
15. Use or overdose of medications	Neuromuscular blocking agents, acetaminophen, opioids, etc.
16. CPR prior to ICU	Whether cardiopulmonary resuscitation was done prior to admission.
17. CPR outcome	If cardiopulmonary resuscitation was done, whether a full resuscitation status was achieved.
18. Surgery prior to ICU	Whether surgical or not. If surgery was done, the anatomical site of surgery.
19. Surgery scheduling	If surgery was done, whether scheduled/elective or emergency.
20. Acute infection prior to ICU	Acute infection prior to ICU admission. Where present, whether nosocomial or not, and the system affected.
21. Culture results	Antimicrobial culture results and sensitivity patterns prior to ICU admission.
22. Antimicrobial agents prior to the ICU	Use of antimicrobial agents prior to ICU admission. If yes, specify dosing.
23. Other therapies prior to the ICU	Use of major therapeutic options prior to ICU admission (including inotropes and vasopressors). If yes, specify dosing.
24. Admission diagnosis and systems affected	The admission diagnosis and specific systems affected (cardiovascular, respiratory, digestive, neurologic, metabolic, hematologic, genitourinary, sepsis, trauma, and miscellaneous) • Cardiovascular: acute myocardial infarction (and anatomical location), unstable angina, chest pain (rule out MI), cardiac arrest, cardiogenic shock, cardiomyopathy, congestive heart failure, hypertension, hypovolemia, hemorrhage, acute aneurysm, peripheral vascular disease, rhythm disturbance, cardiac drug toxicity, others. • Respiratory: airway obstruction, restrictive diseases, asthma, aspiration pneumonia, bacterial pneumonia, viral pneumonia, parasitic/fungal pneumonia, COPD, pleural effusion, edema (noncardiac), embolism, respiratory arrest, cancer (lung, ENT). • Digestive: upper GI bleeding, lower GI bleeding, variceal bleeding, inflammatory diseases, neoplasm, obstruction, perforation, vascular insufficiency, hepatic failure, intra/retroperitoneal bleeding, pancreatitis, others. • Neurologic: intracerebral hemorrhage, neoplasm, infection, neuromuscular disease, drug overdose, subdural/epidural hematoma, subarachnoid hemorrhage, aneurysm, seizure, stroke, others. • Metabolic: acid-base disorders, electrolyte disorders, diabetic ketoacidosis, hyperosmolar diabetic coma, other. • Hematologic: coagulopathy, neutropenia, thrombocytopenia, pancytopenia, others. • Genitourinary: renal, other. • Sepsis: cutaneous, gastrointestinal, pulmonary, urinary, others, unknown • Trauma: head with chest/abdomen/pelvis/spine, head with face/extremity, head only, head with multitrauma, chest with spine, chest alone, spine only, multitrauma (no head), others • Miscellaneous: specify
25. Physiological measurements	Baseline values and trends of core and peripheral body temperature, blood pressures (invasive and non-invasive systolic, diastolic, and pulse pressures), heart rate, respiratory rate, peripheral capillary oxygen saturation, urine output, and Glasgow coma scale scores (and its individual components, i.e. eyes, verbal, and motor)/level of consciousness or new confusion.
26. Laboratory measurements	Baseline values and trends of complete blood count (including hemoglobin concentration, white blood cell count, kidney function tests (including blood urea, creatinine, and urinalysis), liver function tests (including bilirubin and albumin concentrations), clotting profile (including international normalized ratio and activated partial thromboplastin time), blood glucose, HbA1c, and blood electrolytes (including sodium, potassium, calcium, and magnesium).
27. Arterial blood gases	Baseline values and trends of arterial blood gas testing (including arterial partial pressures of oxygen and carbon dioxide, pH, lactate, and base excess) on and off mechanical ventilation and the fraction of inspired oxygen.
28. Measures of infection/inflammation	Baseline results/values and trends of arterial antimicrobial cultures and measures of infections or inflammation (including procalcitonin and C-reactive protein).
29. Mechanical ventilation	Requirement for mechanical ventilation (invasive or non-invasive) and mode of ventilation.
30. Parenteral nutrition	Requirements for parenteral nutrition and the type administered.
31. Thrombolysis	Requirement for thrombolysis.
32. Other relevant information	

Definition of Abbreviations: AIDS, acquired immunodeficiency syndrome; COPD, chronic obstructive pulmonary disease; ENT, ear, nose, and throat; GI, gastrointestinal; ICU, intensive care unit.

In anticipation of building customized equations to ensure better predictive power compared with the existing PSS, additional physical measurements, as well as biological sample collection for measurements of biomarkers, will be done. Urine samples will be taken for assessment of albuminuria based on the KDIGO guidelines [21]. Venous blood samples will be taken for measurement of full blood count, inflammatory biomarkers (such as procalcitonin and C-reactive protein), liver function testing, blood urea, creatinine, electrolyte measurement, and HbA1c concentration [22,23]. Blood and urine samples will be stored for future analyses, following approval by the ethics committees. BMI will not be measured because of feasibility – the beds available in the KBTH ICUs cannot measure weight. However, other anthropometric measures, including mid-upper arm circumference, waist circumference (WC) and waist to hip ratio (WHR) will be measured. WC will be measured with the SECA 201 Girth Circumference Measuring Tape (cm) at the midpoint between the lower margin of the last palpable rib and the iliac crest. WC will be measured in the lying position of expiration (when the lungs are at their functional residual capacity). Hip circumference (HC) will be measured with the SECA 201 Girth Circumference Measuring Tape (cm) at the widest point at the level of the greater trochanters. WHR will be determined as the ratio of WC to HC and will be used to define body fat distribution and abdominal obesity.

### Patient involvement

Study participants were not involved in the design of this study. However, before the commencement of the prospective data collection component of the study, previous ICU survivors, adequately capacitated patients currently in the ICUs, and surrogate decision-makers will be asked to find out their preferences concerning the study procedures. Where applicable, their recommendations will be factored into revising the study procedures after the institutional review boards/ethics committees have been notified.

### Safety considerations

The team of investigators, including trained researchers and specialist/consultant anesthesiologists and intensive care physicians, has the necessary competencies for conducting this study, including being well-versed in clinical research ethics and keenly aware of the responsibility carried by everyone involved in this study. All staff involved in the study have been trained in research involving the critically ill. The PI, anesthesiologists/intensive care physicians, and co-PIs of the study will be responsible for overseeing participant recruitment and study-related clinical procedures. The collection of blood samples will be done by anesthesiologists/intensive care physicians/critical care nurses on the team. During blood sample collection, appropriate steps will be taken to ensure that only minimal pain is caused

### Time plan

Collection of relevant data using the clinical abstraction form will start in June 2025 and is expected to be completed in six months. Recruitment of study participants for the prospective data collection arm will start in June 2025 and is expected to be completed in six months. We anticipate completing the study on January 31, 2026

### Data analysis plan

Data will be analyzed using recent versions of the IBM Statistical Package for the Social Sciences (SPSS) and R. Patients with erroneous/missing prediction score components and/or missing survival data will be excluded when assessing the specific ICU PSSs. Simple tables, proportions, mean, and median values will be used to examine the data. Both graphical methods (including histograms and Q-Q plots) and formal statistical tests (including the Kolmogorov-Smirnov and Shapiro-Wilk tests) will be used to assess the normality of data. Data with normal distribution will be presented as mean (standard deviation), whereas those not normally distributed will be presented as median (interquartile range). Categorical data will be presented as frequency (percentage).

The appropriate ICU PSS scores and their corresponding predicted mortality ratios will be calculated. Observed-to-predicted (expected) mortality rates will be assessed. Validation will be tested by assessing calibration and discrimination. Discrimination (the power to distinguish between non-survivors and survivors) will be determined by the Area Under Receiver Operating Characteristic (AUROC) curve. The outcomes of interest will be the ICU mortality and LOS. The predictors will be the relevant ICU PSS scores. A two-tailed *p*-value < 0.05 will be considered statistically significant. Calibration will be verified using calibration curves and the Hosmer-Lemeshow goodness-of-fit test, and appropriate chi-squared values will be calculated. Calibration curves will be drawn by plotting predicted against actual mortality for groups of the patient population stratified by 10% increments of predicted mortality. We will verify whether the results of the tests of discrimination and calibration differ by relevant factors such as sex, phase of data collection (pre-COVID-19 and post-COVID-19), or sepsis status. If significant interaction is found, the analyses will be stratified accordingly.

Six typically used supervised ML algorithms, namely random forests, gradient boosting, support vector regression, linear regression, multi-layer perceptron, and stacking regression, will be used to identify predictors of ICU mortality. To train the ML model, the entire dataset will be split into a 70% training and 30% testing dataset. To avoid a situation where the algorithm fails to predict anything informative on unseen data, a 5-fold cross-validation on the training dataset for each model will be performed, and their cross-validation performance evaluated. The training model will then be tested on the testing dataset. The mean absolute error and the coefficient of determination (R^2^) will be used to compare the performance across all the models.

### Ethics and dissemination

The study is approved by the Institutional Review Board of the KBTH (Number: KBTH-IRB 000135/2022) and the University of Ghana College of Health Sciences Ethical and Protocol Review Committee (CHS-Et.M.1-P4.13/2023-2024). Further, the study will be conducted in conformity with the current (2024) version of the Helsinki Declaration on Human Experimentation. Only persons meeting the eligibility criteria will be recruited for the study.

For the prospective data collection component, informed consent will be obtained. Critically ill patients are a vulnerable population. To avoid exploitation, the informed consent process will be as follows:

• **Assessment of decision-making capacity**: In the acute phase of the critical illness, the patient may not be adequately capacitated to provide valid consent for the study. Therefore, we will seek consent from a surrogate decision-maker, who will be a family member involved in the admission and care process to the ICU. Some of the patients may regain the capacity to o provide valid consent as their illness improves and/or the effects of medications wear off. Therefore, an assessment of decision-making capacity will be done in the course of the ICU stay. When a patient regains the capacity to make decisions, informed consent will be sought from him or her. His or her wishes will supersede the wishes of any surrogate or family member who gave prior consent.
• **Discussion of study procedures:** The PI or a co-investigator will engage the patient or surrogate decision-maker on the purpose, interventions, potential benefits and risks, and voluntary nature of the research. It will be emphasized that all the interventions (invasive monitoring, mechanical ventilation, arterial and venous blood sampling) are routine in the ICU, and the decision to initiate each of these is solely that of the intensivist taking care of the patient. This study will not warrant an intervention that will otherwise not be instructed by the intensivist or critical care physician. The discussions will be concise, using terminology and languages that the patient or surrogate decision-maker can understand.
• **Comprehension Assessment:** After allowing the patient or surrogate-decision maker to ask questions, the investigator will ask the patient or surrogate-decision maker to summarize the information that has been conveyed. If the summary is deemed adequate by the investigator, comprehension will be assumed to be adequate.
• **Voluntary consenting**: In addition to communicating the study procedures, potential benefits, risks, anonymity, and confidentiality, it will be emphasized that participation in this study is voluntary. The patient or surrogate decision maker will be informed about the freedom to decline to participate or withdraw from the study at any time without penalty or effect on clinical care in the ICU or after discharge from the ICU or other units in the hospital. It will be emphasized that refusal to participate will not affect clinical management in any way.
• **Documentation**: After giving appropriate information about the study, answering all questions, confirming comprehension, and confirming that the patient or surrogate decision-maker has volunteered to take part in the study, the consent form will be administered for signing or thumb printing. A copy of the consent form will be kept in a file, and additional copies will be offered to the study participant or surrogate decision-maker.

All the interviews will take place in the ICU (for patients) or private locations around the ICU (for surrogate decision-makers)

### Patient involvement

• Patients or the public were not involved in the design, conduct, reporting, or dissemination plans of our research

### Protocol validation

None

### Limitations

This study is unable to assess post-hospitalization mortality, as data on this is not available.

## CRediT author statement

**Charles Frederick Hayfron-Benjamin**: Conceptualization, Investigation, Methodology, Project administration, Writing – original draft, Writing – review & editing; **Theresa Ruby Quartey-Papafio**: Investigation, Writing – original draft, Writing – review & editing; **Akua Kissi-Prah**: Investigation, Writing – original draft, Writing – review & editing; **Delphine Delali Grant** Investigation, Writing – original draft, Writing – review & editing; **Tracy Amo-Nyarko**: Investigation, Writing – original draft, Writing – review & editing; **Anastasia Naa Koshie**: Investigation, Writing – original draft, Writing – review & editing; **Divine Agbenyegah Kwami**: Conceptualization, Investigation, Methodology, Writing – original draft, Writing – review & editing; **Andrew Kwabena-Adade**: Conceptualization, Investigation, Methodology, Writing – original draft, Writing – review & editing.

## Supplementary material and/or additional information [OPTIONAL]

None

## Declaration of competing interest

The authors declare that they have no known competing financial interests or personal relationships that could have appeared to influence the work reported in this paper.

## Data Availability

Data will be made available on request.
